# Comparison of speckle-tracking echocardiography with invasive hemodynamics for the detection of characteristic cardiac dysfunction in type-1 and type-2 diabetic rat models

**DOI:** 10.1186/s12933-017-0645-0

**Published:** 2018-01-16

**Authors:** Csaba Mátyás, Attila Kovács, Balázs Tamás Németh, Attila Oláh, Szilveszter Braun, Márton Tokodi, Bálint András Barta, Kálmán Benke, Mihály Ruppert, Bálint Károly Lakatos, Béla Merkely, Tamás Radovits

**Affiliations:** Experimental Research Laboratory, Heart and Vascular Center, Semmelweis University, Városmajor u. 68., 1122 Budapest, Hungary

**Keywords:** Speckle-tracking echocardiography, Invasive hemodynamics, Diabetic cardiomyopathy, Cardiac dysfunction, Heart failure, Murine models, HFpEF, HFrEF

## Abstract

**Background:**

Measurement of systolic and diastolic function in animal models is challenging by conventional non-invasive methods. Therefore, we aimed at comparing speckle-tracking echocardiography (STE)-derived parameters to the indices of left ventricular (LV) pressure–volume (PV) analysis to detect cardiac dysfunction in rat models of type-1 (T1DM) and type-2 (T2DM) diabetes mellitus.

**Methods:**

Rat models of T1DM (induced by 60 mg/kg streptozotocin, n = 8) and T2DM (32-week-old Zucker Diabetic Fatty rats, n = 7) and corresponding control animals (n = 5 and n = 8, respectively) were compared. Echocardiography and LV PV analysis were performed. LV short-axis recordings were used for STE analysis. Global circumferential strain, peak strain rate values in systole (SrS), isovolumic relaxation (SrIVR) and early diastole (SrE) were measured. LV contractility, active relaxation and stiffness were measured by PV analysis.

**Results:**

In T1DM, contractility and active relaxation were deteriorated to a greater extent compared to T2DM. In contrast, diastolic stiffness was impaired in T2DM. Correspondingly, STE described more severe systolic dysfunction in T1DM. Among diastolic STE parameters, SrIVR was more decreased in T1DM, however, SrE was more reduced in T2DM. In T1DM, SrS correlated with contractility, SrIVR with active relaxation, while in T2DM SrE was related to cardiac stiffness, cardiomyocyte diameter and fibrosis.

**Conclusions:**

Strain and strain rate parameters can be valuable and feasible measures to describe the dynamic changes in contractility, active relaxation and LV stiffness in animal models of T1DM and T2DM. STE corresponds to PV analysis and also correlates with markers of histological myocardial remodeling.

**Electronic supplementary material:**

The online version of this article (10.1186/s12933-017-0645-0) contains supplementary material, which is available to authorized users.

## Introduction

Diabetes mellitus (DM) can be divided into four major types including type-1 (T1DM) and type-2 DM (T2DM). T1DM accounts for 5% of all diabetes cases, while T2DM is more common [1]. The number of diabetic adults is likely to increase by 69% in developing countries and 20% in the developed countries by the year of 2030 [2]. DM acts as an independent risk factor for different cardiovascular disorders. The altered metabolism in DM leads to the development of diabetic cardiomyopathy [3, 4]. Many animal models have been developed throughout the recent decades for T1DM and for T2DM to investigate the pathophysiology and pharmacology of diabetic cardiomyopathy [5]. Our research group published a detailed report on characteristic deterioration of cardiac function in T1DM and T2DM animal models [6]. According to our and others’ findings, T1DM is associated with the impairment of contractility and active relaxation while in T2DM increased cardiac stiffness and diastolic dysfunction develops [6–8].

Pressure–volume (PV) analysis is the gold standard method for the detailed hemodynamic characterization of the heart [9]. PV analysis provides the possibility to measure intrinsic cardiac function including contractility, diastolic function, active relaxation and cardiac stiffness independently of loading conditions [10]. In spite of its clear advantages, the greatest disadvantage of PV analysis is its invasiveness (direct catheterization of the heart) and that this method requires the euthanization of the animal.

Transthoracic echocardiography may be an alternative approach and due to its non-invasive nature it may be used in follow-up studies as well. However, conventional parameters derived from two-dimensional and M-mode echocardiographic images suffer from several shortcomings, including modest sensitivity and technical limitations [9, 11]. Thus, assessment of contractility and diastolic function in diabetic cardiomyopathy is an issue for experimental studies. Parameters that are sensitive, reliable and reflect better the systolic and diastolic function of the heart are needed. Speckle-tracking echocardiography (STE) is an emerging echocardiographic technique that allows to characterize the deformation of the myocardium yielding advanced measures of systolic and also diastolic function [9, 12].

Based upon that, in the current study we aimed at comparing STE with PV analysis for the detection of characteristic differences between T1DM- and T2DM-associated cardiac dysfunction in relevant rodent models.

## Methods

### Animals

Animals were housed individually in a room with constant temperature and 12/12 h light/dark cycles.

### Model of T1DM

For T1DM 8-week-old male Sprague–Dawley rats (Charles River, Sulzfeld, Germany) were used. Animals received standard laboratory rat diet and water ad libitum. T1DM was induced with a single i.p. injection of streptozotocin (60 mg/kg) (Sigma Aldrich, Budapest, Hungary). Streptozotocin was dissolved in citrate buffer (pH = 4.5; 0.1 mol/l). Control animals received only the buffer. 72 h after the injection blood glucose concentration was determined by using a digital blood glucose meter (Accu-Chek^®^ Sensor, Roche, Mannheim, Germany). Animals with a random blood glucose level > 15 mmol/l were considered diabetic and included in the study. The following study groups were established: non-diabetic control animals (T1DM Co; n = 5) and animals with type-1 DM (T1DM; n = 8). Experiments were performed after 8 weeks of diabetes duration.

### Model of T2DM

For T2DM 7-week-old Zucker Diabetic Fatty (ZDF) rats (Charles River) were used [6]. Animals develop T2DM due to a genetic mutation of the leptin receptor. Homozygous recessive males (fa/fa) develop obesity, fasting hyperglycemia and T2DM. Homozygous dominant (+/+) and heterozygous (fa/+) lean genotypes remain normoglycemic. Animals were fed a special diet (Purina #5008) and water ad libitum. Animals were assigned to the following groups: non-diabetic control lean animals (T2DM Co; n = 8) and animals with type-2 DM (T2DM; n = 7). Experimental procedures were performed at the age of 32 weeks (after 25 weeks of diabetes duration).

### Measurement of blood glucose

Non-fasting blood glucose was measured by a digital blood glucose meter (Accu-Chek^®^ Sensor, Roche, Mannheim, Germany) from a drop of blood collected from the saphenous vein at the end of the study period.

### Echocardiography

Echocardiography was performed at the end of the experimental period as described elsewhere [13]. In brief, animals were anesthetized with 1–2% isoflurane in 100% oxygen, placed on a heating pad (body temperature was maintained at 37 °C). Echocardiography was conducted by using a 13-MHz linear transducer (GE 12L-RS, GE Healthcare, Horten, Norway) coupled with an echocardiography machine (Vivid *i*, GE Healthcare). Images were analyzed with an image analysis software (EchoPAC v113, GE Healthcare). Long-axis and mid-papillary level short-axis B-mode images were used to measure left ventricular (LV) anterior (AW), posterior wall (PW) thicknesses and LV internal diameter (ID) in end-diastole (d) and in end-systole (s). End-systole was defined at minimal, while end-diastole was defined at maximal LVID. Heart rate was determined from M-mode images taken at the mid-papillary level in the short-axis view of the LV. Values of three consecutive cycles were averaged and used for statistical analysis. LV mass was calculated by the following equation: LVmass = 1.04 × [(LVAWd + LVIDd + LVPWd)^3^ − LVIDd^3^]. LVmass was normalized to the tibia length and body weight [LVmass/tibia length (g/cm); LVmass/body weight]. Fractional shortening (FS) was calculated as FS = (LVIDd − LVIDs)/LVIDd * 100.

### Speckle-tracking echocardiography

**Fig. 1:**
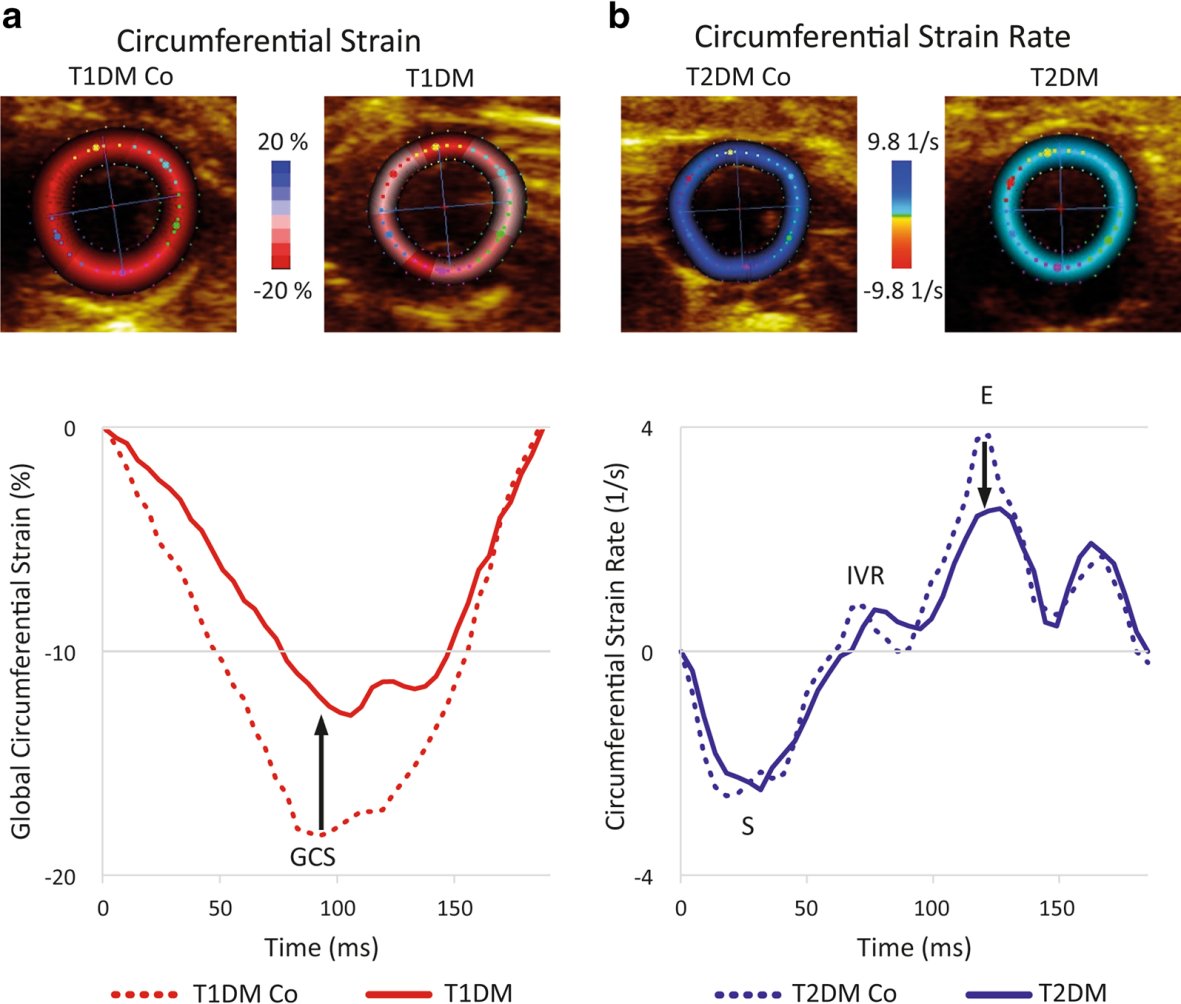
Layout of speckle-tracking analysis. **a** Determination of circumferential strain on short-axis recordings. The negative peak of the curve represents the global circumferential strain (GCS). **b** Determination of circumferential strain rate on short-axis images. The peak negative value is the global circumferential strain rate in systole (SrS). The following peak represents the value of isovolumic relaxation (SrIVR) while the positive peak value is the early diastolic strain rate (SrE). Groups: animals with T1DM, their non-diabetic controls (T1DM Co); animals with T2DM and their non-diabetic controls (T2DM Co). Arrows indicate the specific changes of strain and strain rate curves in DM

Loops of short-axis views of the mid-papillary level dedicated for speckle-tracking analysis were acquired at least three times using a constant frame rate (218 Hz). Analysis was performed in a blinded fashion by an experienced operator (EchoPAC v113). Segmental and global circumferential strain (GCS), circumferential systolic (SrS), isovolumic (SrIVR) and early diastolic (SrE) strain rate values were calculated (Fig. 1). Segmental and global radial strain (GRS) was also measured. Three different short-axis loops from each animal and three cardiac cycles from each loop were analyzed. After manual delineation of the endocardial border on end-diastolic frame, the software automatically divided the region of interest to six segments and tracked them throughout the cardiac cycles. In case of low tracking fidelity, manual correction was performed. Acceptance of a certain segment was guided by the recommendation of the software. Ideally, for each parameter (3 × 3 × 6) 54 segmental values are available. Based on our internal protocol [9], animals with < 36 segmental values available should not be included in statistical analysis. There was no such dropout in the current experiment.

### Hemodynamic measurements

Invasive hemodynamic investigation was performed as described previously [14]. A pressure-conductance microcatheter (SPR-838, Millar Instruments, Houston, TX, USA) was advanced into the LV. In case of T1DM, animals were anesthetized with pentobarbital sodium (60 mg/kg i.p.), while T2DM animals were anesthetized with isoflurane (1–2%). Systolic, diastolic and mean arterial blood pressure (MAP), heart rate, LV end-systolic, end-diastolic pressures and volumes, stroke volume, ejection fraction (EF), cardiac output, stroke work, arterial elastance (Ea), maximal slope of diastolic pressure increment (dP/dt_max_), maximal slope of diastolic pressure decrement (dP/dt_min_), time constant of LV pressure decay (Tau_G_; Glantz-method), maximal power were calculated. The slope of the LV end-systolic PV relationship (ESPVR; linear), preload recruitable stroke work (PRSW), maximal elastance (E_max_) and dP/dt_max_—end-diastolic volume relationship were used as load- and heart rate-independent indices of LV contractility. The slope of the LV end-diastolic PV relationship (EDPVR; linear) was determined as an index of LV diastolic stiffness. A special PV analysis software (PVAN, Millar Instruments) was used for data analysis. Heart weight (g) was measured after euthanization.

### Histology

Five µm thin myocardial sections were stained with Masson’s trichrome and hematoxylin–eosin staining. Interstitial fibrosis (excluding vascular spaces) was evaluated on Masson’s trichrome stained sections. Briefly, fibrotic to total tissue area of the myocardium was determined by ImageJ software (NIH, Bethesda, MD, USA) using thresholding on red–green–blue stack images of Masson’s trichrome sections by two independent observers. Cardiomyocyte hypertrophy was evaluated by the measurement of cardiomyocyte diameter on hematoxylin–eosin stained sections as described previously [14].

### Statistics

Data are presented as mean ± SEM. Shapiro–Wilk test was used to confirm normal distribution. Based on that, unpaired two-sided Student’s *t* test or Mann–Whitney U test was used to compare experimental groups to the corresponding control groups. Pearson or Spearman test was used for correlation analysis appropriately. Individual data of the diabetic animals were normalized to the mean of their corresponding control group. Diabetes associated percent changes were derived and compared with unpaired two-sided Student’s *t* test to compare changes between the two models. To test intra- and interobserver variability of STE measurements, 3 animals from each group were randomly selected and analyzed again using the aforementioned protocol by the first and also by a second operator blinded to previous results. Lin’s concordance correlation coefficient values were calculated. A p < 0.05 was considered significant.

## Results

### Body weight and blood glucose

In T1DM diabetic animals showed significant decrease in body weight, while in T2DM it remained unchanged (Table 1). Blood glucose levels were increased in both DM (T1DM Co vs. T1DM: 7.6 ± 0.4 vs. 33.1 ± 0.2 mmol/l, p < 0.001; T2DM Co vs. T2DM: 9.8 ± 0.6 vs. 27.0 ± 0.6 mmol/l, p < 0.001).

**Table 1:** Basic characteristics and conventional echocardiographic parameters of the study groups

Variable	T1DM Co (n = 5)	T1DM (n = 8)	T2DM Co (n = 8)	T2DM (n = 7)
Body weight (g)	529 ± 13	288 ± 23*	415 ± 9	394 ± 17
Heart weight (g)	1.50 ± 0.05	1.16 ± 0.09*	1.45 ± 0.02	1.48 ± 0.06
Heart weight/body weight (kg/g)	2.8 ± 0.1	4.0 ± 0.1*	3.5 ± 0.1	3.8 ± 0.2
Heart weight/tibia length (g/cm)	0.32 ± 0.01	0.28 ± 0.02	0.35 ± 0.01	0.37 ± 0.01*
Heart rate (beats/min)	364 ± 21	250 ± 10*	300 ± 6	292 ± 6
Fractional shortening (%)	51 ± 3	43 ± 2*	40 ± 2	42 ± 2
LVAWs (mm)	3.3 ± 0.9	2.5 ± 0.9*	2.5 ± 0.9	2.8 ± 0.9
LVAWd (mm)	2.2 ± 0.9	1.5 ± 0.9*	1.7 ± 0.9	1.9 ± 0.9*
LVIDs (mm)	3.0 ± 0.9	3.9 ± 1.0*	4.8 ± 1.0	4.7 ± 1.0
LVIDd (mm)	6.1 ± 1.0	6.9 ± 0.9*	8.1 ± 1.0	8.1 ± 1.0
LVPWs (mm)	3.5 ± 1.0	2.6 ± 0.9*	2.7 ± 0.9	3.2 ± 0.9*
LVPWd (mm)	2.4 ± 0.9	1.7 ± 0.9*	1.7 ± 0.9	2.2 ± 0.9*

The table shows the following parameters in the experimental groups: body weight, heart weight, heart weight/body weight, heart weight to tibia length, blood glucose, heart rate (during echocardiography), fractional shortening, left ventricular (LV) anterior (AW) and posterior wall (PW) thicknesses in systole (s) and in diastole (d), LV internal diameter (ID) in ‘s’ and ‘d’. Groups: animals with T1DM, their non-diabetic controls (T1DM Co); animals with T2DM and their non-diabetic controls (T2DM Co). * p < 0.05 vs. corresponding control group

### Echocardiography

T1DM was associated with significantly decreased wall thicknesses in diastole and in systole while LVIDd and LVIDs showed significant increase compared to controls (Table 1). In spite of that, in LVAWd and LVPW values showed marked increase in T2DM animals in comparison to controls (Table 1) while LVID values remained unchanged. Additionally, FS decreased significantly in T1DM, while it was preserved in T2DM (Table 1).

### Speckle-tracking echocardiography

**Fig. 2:**
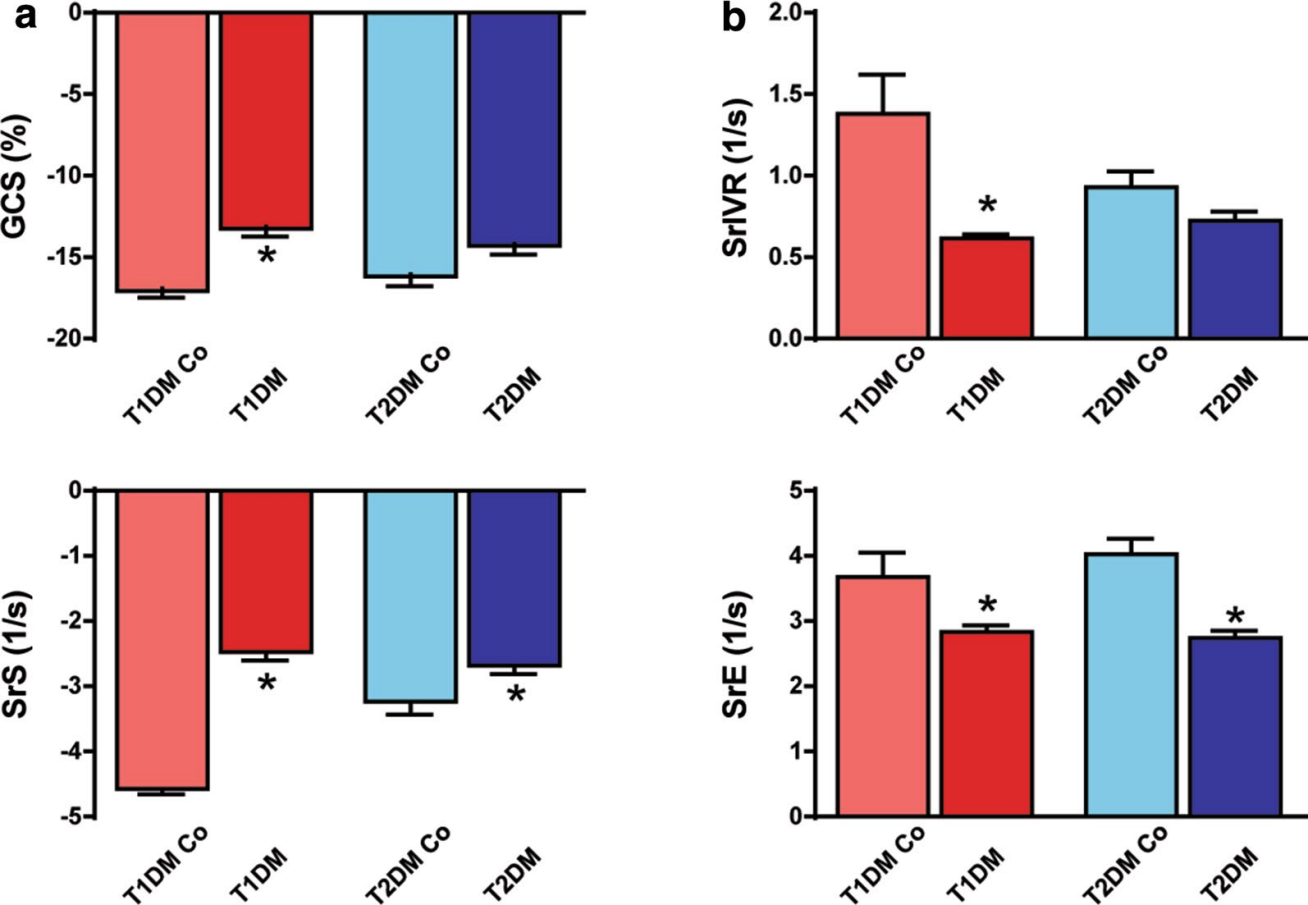
Results of speckle-tracking echocardiography. **a** Graphs show systolic parameters: global circumferential strain (GCS) and peak strain rate values in systole (SrS). **b** Graphs show diastolic parameters: peak strain rate at isovolumic relaxation (SrIVR) and in early diastole (SrE) in the groups. Groups: animals with T1DM, their non-diabetic controls (T1DM Co); animals with T2DM and their non-diabetic controls (T2DM Co). *p < 0.05 vs. corresponding controls

In T1DM animals, GCS and SrS values indicated clear systolic dysfunction compared to controls (Fig. 1 and Fig. 2a). However, in the T2DM group only SrS was slightly, but significantly decreased versus control animals (Fig. 2a). SrIVR (suggested to be correlated with active relaxation) [15] was deteriorated in the T1DM model (Fig. 2b), while SrE was significantly reduced in T1DM and T2DM as well (Fig. 1 and Fig. 2b). GRS, along with segmental strain values, are reported in Additional file 1: Table S1.

### Invasive hemodynamics

**Fig. 3:**
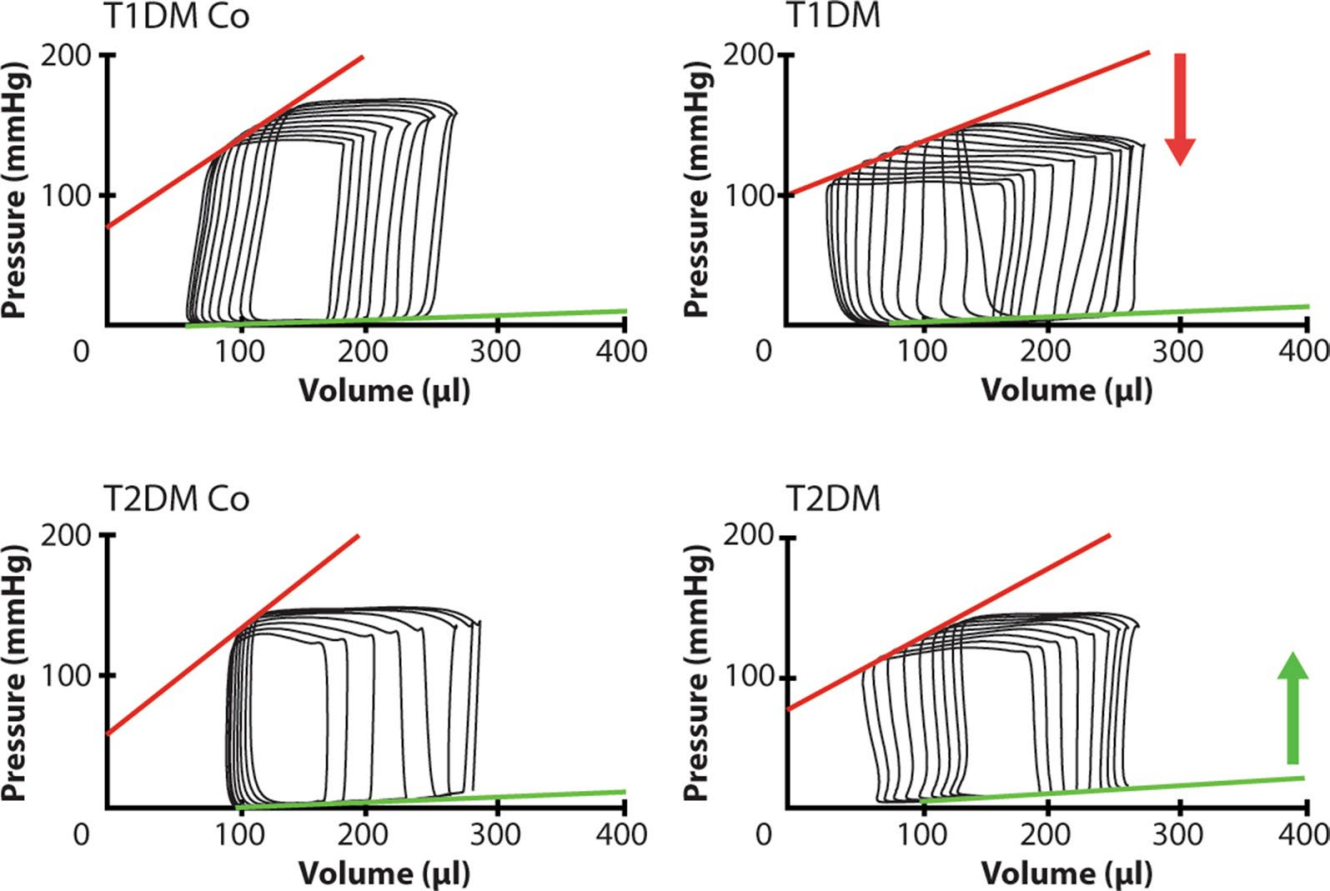
Representative pressure–volume loops. The figure shows representative left ventricular pressure–volume (PV) loops of the animals. Red line: end-systolic PV relationship; green line: end-diastolic PV relationship. Arrows indicate the change of PV relationships (red arrow: decrease in end-systolic PV relationship, green arrow: increase in end-diastolic PV relationship). Groups: animals with T1DM, their non-diabetic controls (T1DM Co); animals with T2DM and their non-diabetic controls (T2DM Co)

T1DM was associated with lower MAP and heart rate along with the significant worsening of contractile function as shown by decreased values of EF, cardiac output, ESPVR and PRSW versus control rats (Fig. 3 and Additional file 1: Table S2). On the other hand, MAP was similar, systolic function was preserved in the animal model of T2DM as represented by unchanged EF, cardiac output and ESPVR (Fig. 3 and Additional file 1: Table S2). PRSW showed a slight decrease in LV contractility in T2DM (Additional file 1: Table S2). On the contrary, we observed significant increase of EDPVR (a marker of cardiac stiffness) in the T2DM rats, while it increased slightly in T1DM (Fig. 3 and Additional file 1: Table S2). Additionally, Tau_G_ was markedly increased in both DM models (Additional file 1: Table S2). However, dP/dt_min_ deteriorated only in T1DM and remained unchanged in T2DM (Additional file 1: Table S2). LVEDP increased only in T2DM (Additional file 1: Table S2). All relevant data generated and calculated by the PVAN software are reported in Additional file 1: Table S2.

### Diabetes associated impairment of cardiac function

**Fig. 4:**
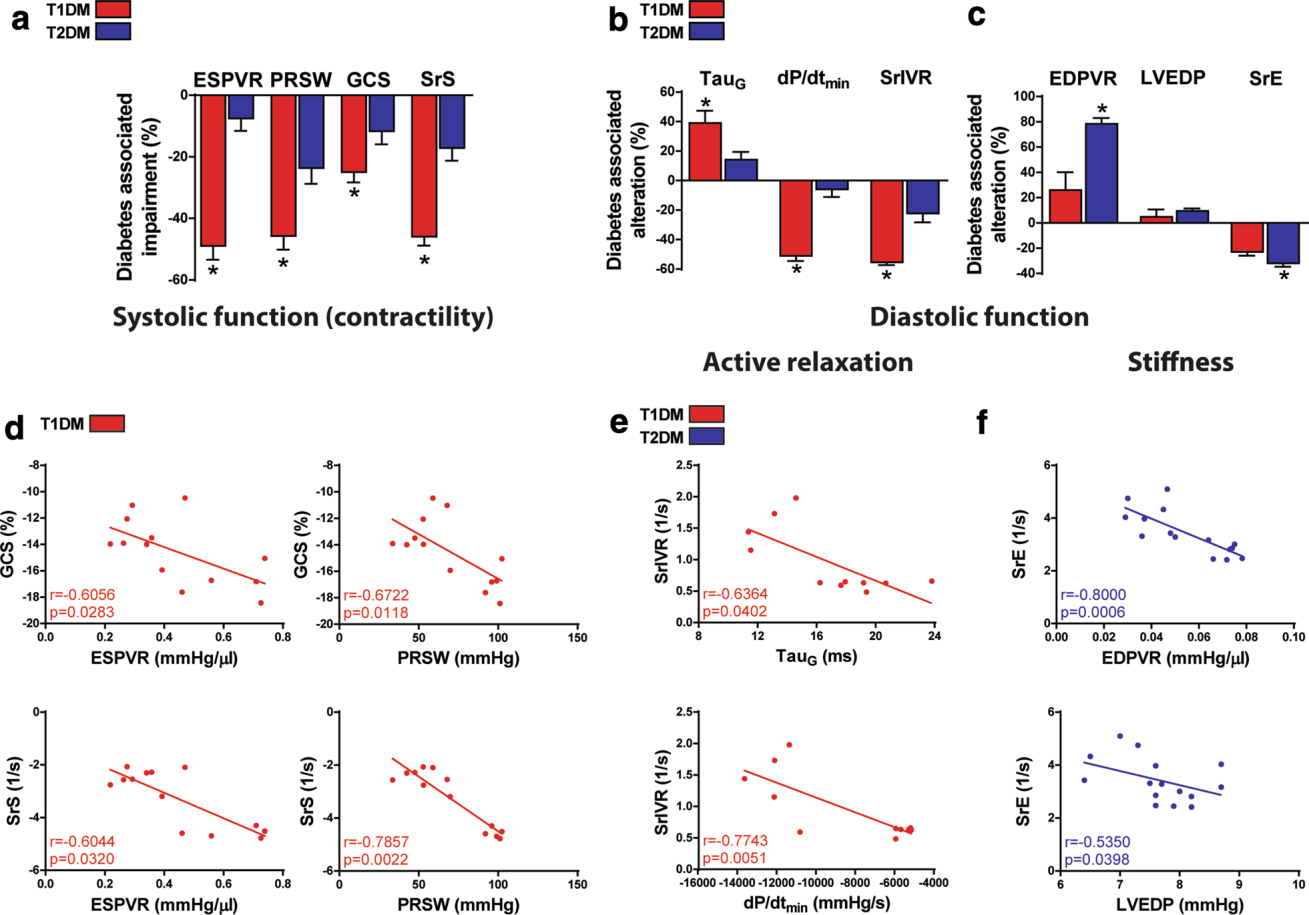
Diabetes associated impairment of systolic and diastolic function in diabetes. **a** Diabetes associated impairment of the following contractile parameters in type-1 and type-2 diabetes mellitus (T1DM, T2DM): end-systolic pressure–volume relationship (ESPVR), preload recruitable stroke work (PRSW), global circumferential strain (GCS) and the peak strain rate values in systole (SrS). **b** Diabetes associated alteration of diastolic parameters Tau_G_, maximal slope of diastolic decrement (dP/dt_min_) and strain rate at isovolumic relaxation (SrIVR) are shown. **c** Diabetes associated alteration of the slope of end-diastolic pressure–volume relationship (EDPVR), left ventricular end-diastolic pressure (LVEDP) and strain rate in early diastole (SrE) are shown. **d** Correlation analyses of GCS and SrS to ESPVR and PRSW are shown. **e** Correlation analyses of active relaxation parameter SrIVR to Tau_G_ and dP/dt_min_ are shown. **f** Correlation analyses of SrE to EDPVR and LVEDP are shown. Groups: animals with T1DM, their non-diabetic controls (T1DM Co); animals with T2DM and their non-diabetic controls (T2DM Co). *p < 0.05 T1DM vs. T2DM

Regarding the cardiac function we observed different pattern of hemodynamic alterations in T1DM and T2DM. Contractile function was more impaired in T1DM as shown by the values of ESPVR and PRSW and by STE parameters GCS and SrS (Fig. 4a). Regarding diastolic function, we observed more severe deterioration of active relaxation in T1DM (Fig. 4b), however, cardiac stiffness was more pronounced in T2DM (Fig. 4c). Accordingly, we observed significant correlations of the parameters of GCS and SrS with the contractile parameters of the PV analysis in T1DM (Fig. 4d; GCS and PRSW r = − 0.672, p < 0.05; SrS and PRSW r = − 0.786, p < 0.01). In addition to that, markers of active relaxation Tau_G_ and dP/dt_min_ correlated with SrIVR in T1DM (Fig. 4e), while the myocardial stiffness parameter EDPVR correlated with SrE in T2DM (Fig. 4f; EDPVR and SrE r = 0.800, p < 0.001).

### EDPVR and SrE correlate with myocardial hypertrophy in T2DM

**Fig. 5:**
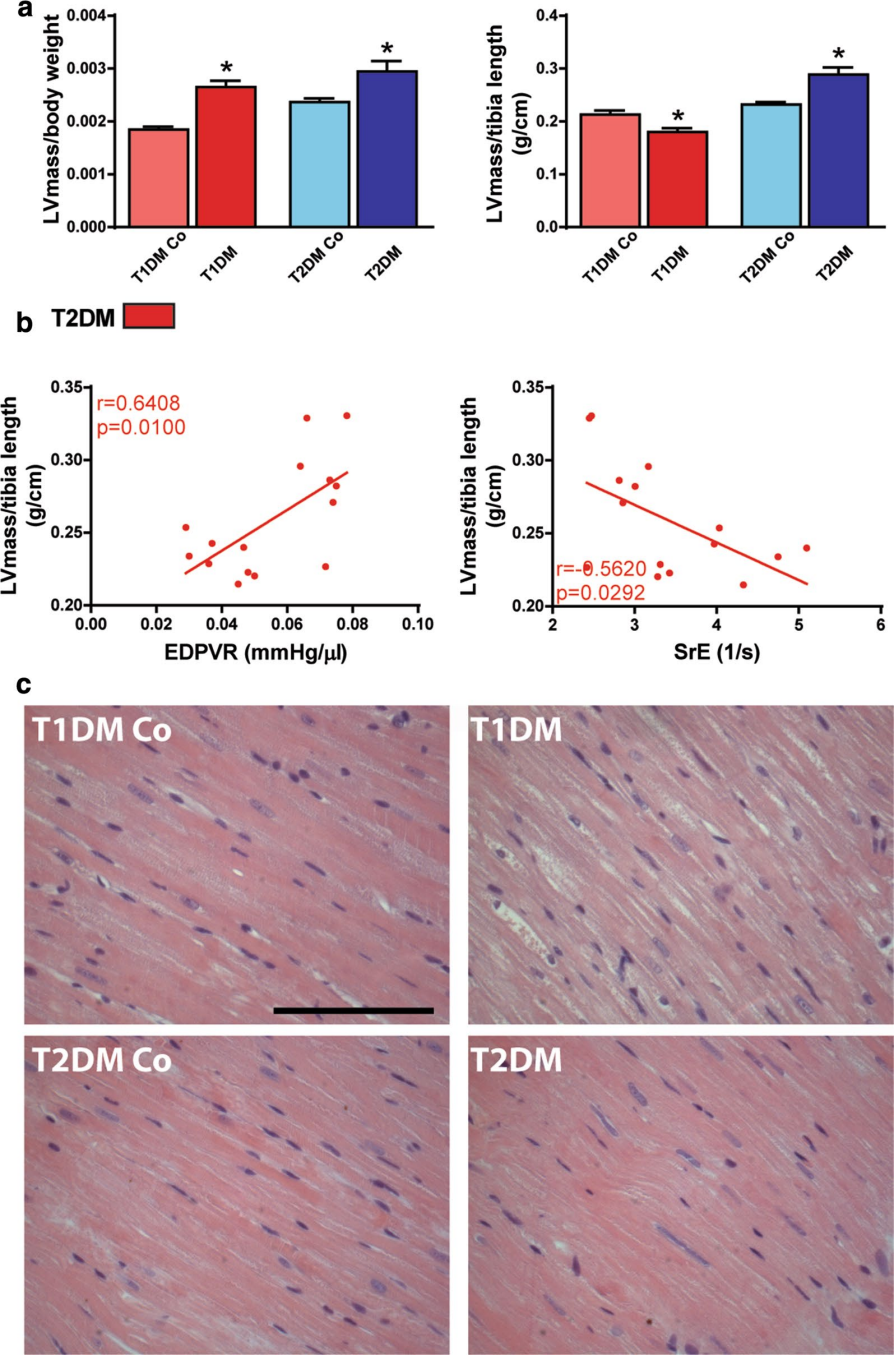
Cardiac stiffness correlates with the extent of myocardial hypertrophy. **a** Graphs of left ventricular (LV) mass to body weight (LVmass/body weight) and to tibia length (LVmass/tibia length) are shown. **b** Results of correlation analysis of LVmass/tibia length to the slope of end-diastolic pressure–volume relationship (EDPVR) and to early diastolic strain rate (SrE). **c** Representative hematoxylin–eosin stained sections. Magnification: ×400. Marker: 100 µm. *p < 0.05 vs. corresponding controls

**Fig. 6:**
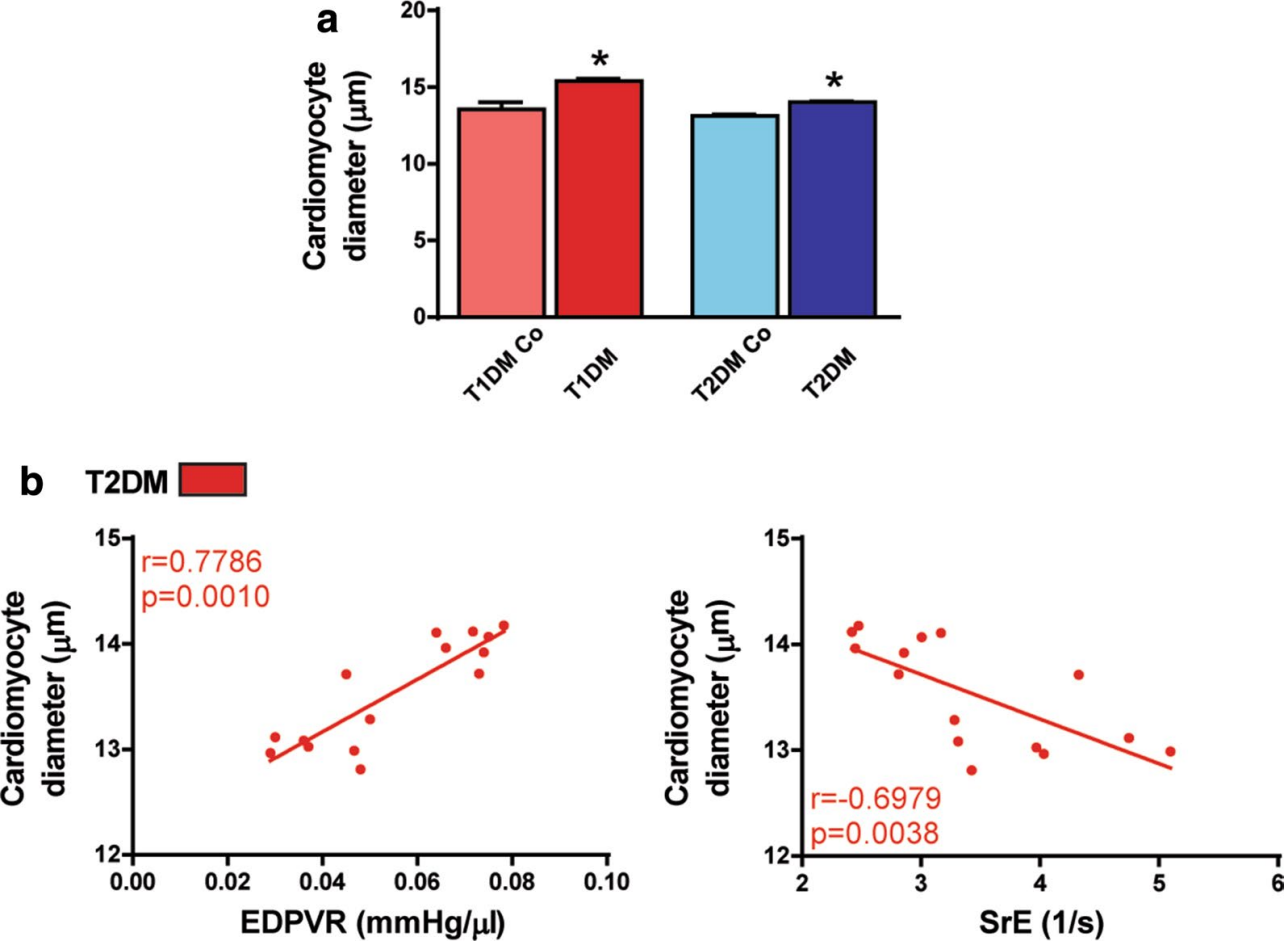
Cardiac stiffness correlates with the extent of myocardial hypertrophy. **a** Analysis of cardiomyocyte diameter in the study groups. **b** Correlation analyses of cardiomyocyte diameter to EDPVR and SrE. Groups: animals with T1DM, their non-diabetic controls (T1DM Co); animals with T2DM and their non-diabetic controls (T2DM Co). *p < 0.05 vs. corresponding controls

Although heart weight was significantly lower in T1DM, the heart weight/body weight ratio was increased in T1DM animals (Table 1). Additionally, these parameters did not change significantly in T2DM (Table 1). However, heart weight/tibia length ratio was increased in T1DM animals when compared to the controls (Table 1). Interestingly, echocardiographic analysis revealed increased LVmass/body weight values in both models (Fig. 5a) while LVmass/tibia length showed significant decrease in T1DM (Fig. 5a) and marked increase in T2DM (Fig. 5a). The sensitive parameter of cardiac stiffness, EDPVR, along with SrE correlated significantly with the extent of myocardial hypertrophy in T2DM. Of note, EDPVR and SrE correlated with the echocardiography-derived LVmass/tibia length ratio (Fig. 5b; EDPVR r = 0.641, p < 0.05; SrE r = − 0.562, p < 0.05). Moreover, histological analysis (Fig. 5c) revealed the significant elevation of cardiomyocyte diameter in both models (Fig. 6a) and EDPVR and SrE showed closer correlations with the hypertrophy of individual cardiomyocytes (represented by cardiomyocyte diameter) on a histological level (Fig. 6b; EDPVR r = 0.779, p < 0.01; SrE r = − 0.698, p < 0.01).

### EDPVR and SrE correlate with myocardial fibrosis in T2DM

**Fig. 7:**
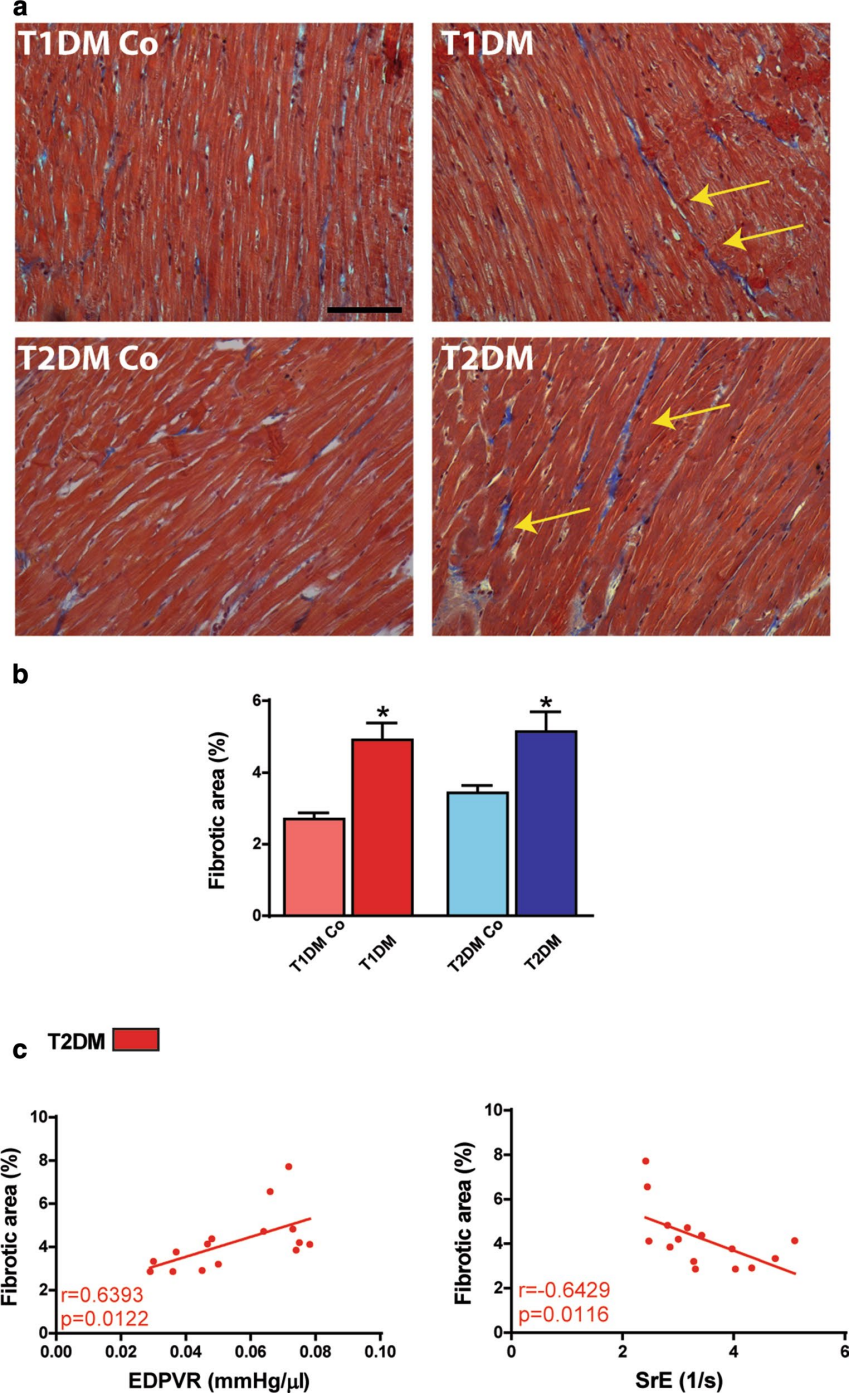
Cardiac stiffness correlates with myocardial fibrosis. **a** Masson’s trichrome stained images. Magnification: ×100. Marker: 100 µm. **b** Quantification of fibrotic area in the study groups. **c** Result of correlation analysis of EDPVR and SrE to fibrotic area. Groups: animals with T1DM, their non-diabetic controls (T1DM Co); animals with T2DM and their non-diabetic controls (T2DM Co). *p < 0.05 vs. corresponding controls

As a sign of myocardial remodeling, fibrosis of the LV was evident in both models (Fig. 7a, b). It is worth noting that not only cardiac hypertrophy but also myocardial fibrosis is a determinant of cardiac stiffness since the values of EDPVR and also STE-derived SrE correlated with the fibrotic area of the LV (Fig. 7c).

### Intra- and interobserver variability of STE parameters

Intraclass correlation coefficient values were the highest in terms of SrS (intra-reader and inter-reader: 0.982 and 0.969, respectively). GCS showed almost no difference between intra- and interobserver variability (0.950 and 0.947). SrE (0.971 and 0.929) and SrIVR (0.969 and 0.922) had similar intra-, while lower interobserver reproducibility. Intraclass correlation coefficient values of GRS were lower (0.876 and 0.811).

## Discussion

In the present study we showed that: (a) STE-derived measurements are sensitive in the detection of diabetes associated impairment of cardiac contractility as well as diastolic function, (b) STE-derived parameters correlate with cardiac contractility, active relaxation and stiffness measured by PV analysis and (c) indices of STE correlate with the markers of histological remodeling (hypertrophy and fibrosis) in T2DM related diabetic cardiomyopathy.

The rat model of streptozotocin-induced T1DM and the ZDF rat as a model of T2DM are widely used in studies focusing on the characterization of diabetic cardiomyopathy. Depending on the type of DM it is associated with characteristic changes in body weight and blood glucose levels. T1DM is associated with insulinopenia and weight loss, while T2DM is often associated with obesity and hyperinsulinemia [1, 16]. In line with these observations, we found increased blood glucose levels in both models, however, body weight was lower in T1DM while it did not change in T2DM.

Characterization of cardiac performance is challenging in DM due to the changes in MAP and heart rate [10]. These phenomena might contribute to the difficulties of the comparison of cardiac function in these models with conventional techniques. Transthoracic echocardiography is the most commonly used imaging modality as it allows detailed morphological and functional characterization of the heart, while still remaining radiation free and cost-effective. However, conventional parameters suffer from several shortcomings including modest sensitivity and reproducibility, resulting in moderate correlations with invasive measurements [11]. Development of software technology permitted frame-by-frame tracking of the myocardial echogenic pattern and this non-Doppler approach defines several advanced parameters referring to systolic and diastolic function [17]. Strain measurements may notably overcome the aforementioned limitations by reflecting intrinsic myocardial function and also by being a semi-automated method. Sensitivity of strain measures were proved in numerous pathological conditions in human and also in animal experiments [18, 19] including diabetic cardiomyopathy [4, 20–23]. Furthermore, in our recent work, we were able to show correlations between STE and PV analysis derived measures in a rat model of increased cardiac contractility [9]. Here, we found that GCS and SrS can demonstrate the overt systolic dysfunction in T1DM and correlate well with invasively measured contractility. However, technical setup of our experiment allows detailed characterization of diastole as well. In our current work, SrIVR was reduced significantly in T1DM, while the deterioration of SrE was more pronounced in T2DM. This parallels with the invasive PV analysis showing more evident deterioration of active relaxation in T1DM along with pronounced increase in LV stiffness in T2DM. Previously, SrIVR has been shown to be associated with Tau_G_ [24]. In our study, this association was also present in T1DM animals. Additionally, a correlation between SrE and the stiffness parameters EDPVR and LVEDP was shown in T2DM. Formerly, decrease of circumferential strain was demonstrated in a mouse model of T2DM [25], while most recently Shepherd et al. published their data on T1DM highlighting the early sensitive nature of STE during consecutive evaluation compared to conventional echocardiographic parameters [26]. Based on these previous results, STE is now increasingly used in human clinical trials to unmask and follow-up subtle alterations in LV function [27]. Moreover, STE is also capable to assess right ventricular and also atrial deformation, which measurements will contribute to a more complex understanding of diabetic cardiomyopathy [28, 29].

Depending on its type, DM is associated with characteristic changes of cardiac function. Diastolic dysfunction is an early sign in DM associated cardiomyopathy before the onset of detectable systolic dysfunction [3]. However, T1DM animals have been shown to exert not only diastolic but systolic cardiac dysfunction already at early time-points during the progression of the disease [3, 16, 26, 30, 31]. According to our previous work, streptozotocin-induced (insulin-dependent) T1DM associated diabetic cardiomyopathy is characterized by the impairment of systolic function and by the prolongation of active relaxation [6]. Contractility and active relaxation are highly energy-dependent processes that are greatly affected by the disturbances of insulin signaling [3]. Accordingly, we observed more pronounced impairment of the energy-dependent measures of systolic function (EF, cardiac output, ESPVR, PRSW; GCS, SrS) and active relaxation (Tau_G_, dP/dt_min_; SrIVR) in our T1DM model by PV analysis and by STE. On the contrary, our T2DM animal model has been shown to develop insulin resistance together with hyperinsulinemia and insulinopenia at a later stage of the disease [32]. Accordingly, systolic function is preserved along with the impairment of diastolic function in the T2DM rat model [6]. Our present results are in line with the above findings as systolic parameters (EF, cardiac output, ESPVR; GCS) did not change significantly in T2DM. However, LV compliance indices were markedly altered (LVEDP, EDPVR) in T2DM, which is in line with our previous results [6, 16]. Additionally, we observed a significant drop in MAP and heart rate in T1DM while they did not change in T2DM. End-diastolic stiffness is predominantly affected by the changes of myocardial structure [33] including cardiomyocyte hypertrophy and fibrotic remodeling [34]. Consistently, we found increased LVEDP and EDPVR in T2DM while SrE was also markedly reduced. In the background of the increased diastolic stiffness we observed hypertrophic and fibrotic remodeling of the myocardium in T2DM. Of note, our sensitive hemodynamic parameter EDPVR along with STE-derived SrE correlated with the extent of cardiomyocyte hypertrophy and with the amount of interstitial fibrosis. Previous studies have also reported that STE is a useful tool in the assessment of myocardial fibrosis as global and segmental strain parameters correlated with interstitial fibrosis and cardiomyocyte area in animal models of uremic [35] and hypertrophic cardiomyopathy [36] as well as in humans with Fabry-disease [37] and hypertrophic cardiomyopathy [38]. However, to our knowledge this is the first study that shows that the impairment of SrE could refer to the extent of cardiac hypertrophy and fibrosis. Additionally, to our knowledge our study firstly reports about the correlation between EDPVR, SrE and histological markers of hypertrophy and interstitial fibrosis.

## Limitations

Our study has some assumptions and limitations. Firstly, the number of investigated animals may serve as a limitation, however, the current case numbers are reasonable in experimental studies and are based on previous statistical calculations (power analysis). This should also affect some of the calculated correlation coefficients, where beyond the peak values, the dispersion of data is higher in the mid-range. While we should be cautious with the interpretation of the exact r values, the presence of a correlation is assured. Second, the echocardiographic hardware and software environment is not dedicated to small animal models. Nevertheless, the correlations with PV analysis, and also the reported intra- and interobserver values suggest that both the sensitivity and the reproducibility of our setup is comparable to dedicated, higher frequency systems [12, 39]. Longitudinal strain, which is the most robust parameter in human settings, is absent in this work due to the lack of sufficient apical window in rats and due to its less reliable measurement from available long-axis images. However, it has been extensively shown that both regional and global STE parameters derived from short-axis recordings could be feasible and meaningful measurements in various pathological conditions and experimental settings [40–42]. Beyond cardiomyocyte diameter, myocardial hypertrophy is also described by the calculation of LV mass, an M-mode measurement relying on geometrical assumptions. The inherent error in the calculation may have caused the modest correlation with stiffness parameters.

## Conclusions

According to our knowledge, this is the first study to compare STE-derived systolic and also diastolic functional measurements with PV analysis in detecting the characteristic differences between T1DM- and T2DM-associated cardiac dysfunction in experimental animal models. Based on our results, strain and strain rate parameters might be valuable and feasible measures to describe the dynamic changes in contractility, active relaxation and LV stiffness in animal models of T1DM and T2DM by serial assessment. Moreover, these parameters may be reliable tools for experimental studies focusing on the pathophysiology and on the monitoring of therapeutic interventions in both type-1 and type-2 DM.

## Additional file

**Additional file 1.** Additional tables.

## Abbreviations

AW: anterior wall
d: diastole
DM: diabetes mellitus
dP/dt_min_: maximal slope of diastolic pressure decrement
EDPVR: the slope of the LV end-diastolic PV relationship
EF: ejection fraction
ESPVR: the slope of the LV end-systolic PV relationship
FS: fractional shortening
GCS: global circumferential strain
GRS: global radial strain
ID: internal diameter
LV: left ventricle
LVEDP: left ventricular end-diastolic pressure
MAP: mean arterial blood pressure
PRSW: preload recruitable stroke work
PV: pressure–volume
PW: posterior wall
s: systole
STE: speckle-tracking echocardiography
SrE: early diastolic strain rate
SrIVR: strain rate at isovolumic relaxation
SrS: peak systolic strain rate
T1DM: type-1 diabetes mellitus
T2DM: type-2 diabetes mellitus
Tau_G_: time constant of LV pressure decay
ZDF: Zucker Diabetic Fatty
