# Supplementary material for: Comparison of speckle-tracking echocardiography with invasive hemodynamics for the detection of characteristic cardiac dysfunction in type-1 and type-2 diabetic rat models
Source: Cardiovasc Diabetol. 2018 Jan 16;17:13. doi: 10.1186/s12933-017-0645-0 (PMC5769218; doi:10.1186/s12933-017-0645-0)
Supplement: Supplementary file 1 — Additional file 1. Additional tables. [file 12933_2017_645_MOESM1_ESM.docx]

**Title: Comparison of speckle-tracking echocardiography with invasive hemodynamics for the detection of characteristic cardiac dysfunction in type-1 and type-2 diabetic rat models**

**Supplementary material**

**Table S1.** Segmental and global circumferential and radial strain values of the study groups assessed by speckle-tracking echocardiography.

| **Circumferential strain** | **T1DM Co (n=5)** | **T1DM (n=8)** | **T2DM Co (n=8)** | **T2DM (n=7)** |
| --- | --- | --- | --- | --- |
| inferoseptal segment circumferential strain (%) | -19.2±3.8 | -14.9±0.8 | -17.5±1.2 | -17.4±1.5 |
| anteroseptal segment circumferential strain (%) | -22.6+1.7 | -10.9±1.9* | -21.4±1.4 | -12.6±2.2* |
| anterior segment  circumferential strain (%) | -21.3±1.2 | -19.3±0.7 | -20.2±2.0 | -15.7±1.7 |
| anterolateral segment circumferential strain (%) | -14.2±1.9 | -9.3±2.4 | -12.1±1.7 | -8.9±4.4 |
| inferolateral segment circumferential strain (%) | -11.2±2.8 | -8.3±1.8 | -12.8±2.2 | -12.2±1.7 |
| inferior segment  circumferential strain (%) | -21.7±1.3 | -20.9±1.5 | -15.7±3.2 | -15.7±1.8 |
| global circumferential strain (%) | -16.9±0.6 | -13.1±0.6* | -16.0±0.7 | -14.2±0.7 |
| **Radial strain** | **T1DM Co (n=5)** | **T1DM (n=8)** | **T2DM Co (n=8)** | **T2DM (n=7)** |
| inferoseptal segment  radial strain (%) | 72.1±5.0 | 40.5±5.4* | 57.1±3.1 | 40.4±1.9* |
| anteroseptal segment  radial strain (%) | 73.6±7.9 | 37.8±4.9* | 59.6±3.4 | 36.2±2.7* |
| anterior segment  radial strain (%) | 59.1±4.1 | 39.0±4.6* | 49.6±4.7 | 36.8±2.7 |
| anterolateral segment  radial strain (%) | 50.9±7.1 | 41.8±4.9 | 51.4±4.6 | 29.2±2.6* |
| inferolateral segment  radial strain (%) | 54.1±6.4 | 41.6±3.8 | 53.2±4.5 | 29.7±1.2* |
| inferior segment  radial strain (%) | 64.8±3.4 | 38.3±4.6* | 53.7±4.4 | 42.2±5.5 |
| global radial strain (%) | 61.5±3.7 | 40.1±3.5* | 54.8±2.8 | 35.9±2.0* |

Groups: animals with T1DM, their non-diabetic controls (T1DM Co); animals with T2DM and their non-diabetic controls (T2DM Co). *p<0.05 vs. corresponding control group.

**Table S2.** Results of pressure-volume analysis of the study groups.

| **Basic parameters** | **T1DM Co (n=5)** | **T1DM (n=8)** | **T2DM Co (n=8)** | **T2DM (n=7)** |
| --- | --- | --- | --- | --- |
| Systolic arterial pressure (mmHg) | 170±7 | 148±5* | 124±4 | 129±2 |
| Diastolic arterial pressure (mmHg) | 136±4 | 113±3* | 85±2 | 85±2 |
| Mean arterial pressure (mmHg) | 153±5 | 127±4* | 98±2 | 100±2 |
| Heart rate (beats/min) | 412±11 | 337±11* | 324±3 | 312±5 |
| LV end-systolic volume (µl) | 99±7 | 253±11* | 123±9 | 119±9 |
| LV end-diastolic volume (µl) | 253±11 | 142±10 | 306±17 | 271±15 |
| LV end-systolic pressure (mmHg) | 179±6 | 127±4* | 113±5 | 114±3 |
| LV end-diastolic pressure (mmHg) | 6.5±0.2 | 6.8±0.4 | 7.3±0.3 | 8.0±0.1* |
| Stroke volume (µl) | 155±8 | 137±12 | 184±17 | 152±7 |
| Ejection fraction (%) | 61±2 | 49±4* | 59±3 | 56±1 |
| Cardiac output (µl/min) | 63523±2736 | 45819±3700* | 59375±5625 | 47592±2485 |
| Stroke work (mmHg*μl) | 23602±1404 | 17344±1569* | 18035±2022 | 15146±574 |
| Arterial elastance (Ea) (mmHg/µl) | 1.17±0.05 | 1.00±0.12 | 0.57±0.04 | 0.70±0.08 |
| dP/dt_max_ (mmHg/sec) | 10538±473 | 8464±636* | 9395±434 | 8462±245 |
| dP/dt_min_ (mmHg/sec) | -12039±474 | -6026±442* | -9912±501 | -9318±508 |
| Tau_G_ (msec) | 13.7±1.2 | 18.7±1.2* | 10.0±0.2 | 11.5±0.5* |
| Maximal power (mWatts) | 126±9 | 63±6* | 90±15 | 82±14 |
| **Sensitive contractility and stiffness parameters** | **T1DM Co (n=5)** | **T1DM (n=8)** | **T2DM Co (n=8)** | **T2DM (n=7)** |
| ESPVR (mmHg/μl) | 0.64±0.06 | 0.33±0.03* | 0.61±0.04 | 0.56±0.02 |
| PRSW (mmHg) | 98±2 | 53±4* | 108±4 | 82±6* |
| EDPVR (mmHg/μl) | 0.038±0.003 | 0.048±0.005 | 0.040±0.003 | 0.072±0.002* |
| E_max_ (mmHg/μl) | 2.47±0.41 | 1.38±0.14* | 1.85±0.12 | 1.61±0.15 |
| dP/dt_max_ - EDV (mmHg/s/ml) | 36.8±4.2 | 19.6±3.1* | 36.6±3.2 | 31.5±4.5 |

Groups: animals with T1DM, their non-diabetic controls (T1DM Co); animals with T2DM and their non-diabetic controls (T2DM Co). Abbreviations: the slope of the end-systolic pressure-volume relationship (ESPVR), the preload recruitable stroke work (PRSW), the slope of end-diastolic pressure-volume relationship (EDPVR), time constant of active relaxation (Tau_G_), maximal slope of diastolic increment and decrement (dP/dt_max_; dP/dt_min_), maximal elastance (E_max_), dP/dt_max_ – end-diastolic volume relationship (dP/dt_max_ – EDV), left ventricle (LV). *p<0.05 vs. corresponding control group.
